# Machine Perfusion of Porcine Livers with Oxygen-Carrying Solution Results in Reprogramming of Dynamic Inflammation Networks

**DOI:** 10.3389/fphar.2016.00413

**Published:** 2016-11-04

**Authors:** David Sadowsky, Ruben Zamora, Derek Barclay, Jinling Yin, Paulo Fontes, Yoram Vodovotz

**Affiliations:** ^1^Department of Surgery, University of Pittsburgh, PittsburghPA, USA; ^2^McGowan Institute for Regenerative Medicine, University of Pittsburgh, PittsburghPA, USA; ^3^Department of Surgery, Thomas E. Starzl Transplantation Institute, PittsburghPA, USA

**Keywords:** modeling, transplant, porcine, inflammation, reprogramming, networks

## Abstract

**Background:** *Ex vivo* machine perfusion (MP) can better preserve organs for transplantation. We have recently reported on the first application of an MP protocol in which liver allografts were fully oxygenated, under dual pressures and subnormothermic conditions, with a new hemoglobin-based oxygen carrier (HBOC) solution specifically developed for *ex vivo* utilization. In those studies, MP improved organ function post-operatively and reduced inflammation in porcine livers. Herein, we sought to refine our knowledge regarding the impact of MP by defining dynamic networks of inflammation in both tissue and perfusate.

**Methods:** Porcine liver allografts were preserved either with MP (*n* = 6) or with cold static preservation (CSP; *n* = 6), then transplanted orthotopically after 9 h of preservation. Fourteen inflammatory mediators were measured in both tissue and perfusate during liver preservation at multiple time points, and analyzed using Dynamic Bayesian Network (DyBN) inference to define feedback interactions, as well as Dynamic Network Analysis (DyNA) to define the time-dependent development of inflammation networks.

**Results:** Network analyses of tissue and perfusate suggested an NLRP3 inflammasome-regulated response in both treatment groups, driven by the pro-inflammatory cytokine interleukin (IL)-18 and the anti-inflammatory mediator IL-1 receptor antagonist (IL-1RA). Both DyBN and DyNA suggested a reduced role of IL-18 and increased role of IL-1RA with MP, along with increased liver damage with CSP. DyNA also suggested divergent progression of responses over the 9 h preservation time, with CSP leading to a stable pattern of IL-18-induced liver damage and MP leading to a resolution of the pro-inflammatory response. These results were consistent with prior clinical, biochemical, and histological findings after liver transplantation.

**Conclusion:** Our results suggest that analysis of dynamic inflammation networks in the setting of liver preservation may identify novel diagnostic and therapeutic modalities.

## Introduction

Organ availability for liver transplantation continues to fall well short of demand. During 2012 in the United States, 6,256 transplants were performed compared with 10,143 candidates added to the waiting list. A troubling trend contributing to this imbalance is the increasing organ discard rate, which has risen from 15% in 2004 to 21% in 2010. Organ discard has been associated with older donor age, higher body mass index, and diabetes, all of which have been increasing in the donor population (Orman et al., 2013).

One strategy to reduce the discard rate is to improve organ preservation from the current standard method of CSP, where organs are stored in hypothermic (4°C) and anoxic conditions. A variety of applications of *ex vivo* MP as a means to better preserve organs have been investigated recently with promising results (Monbaliu and Brassil, 2010). MP is a technology developed to provide better conditions for organ preservation before transplantation (Lindbergh et al., 1966). MP devices are built primarily as closed perfusion systems capable of pumping preservation solutions (perfusate) through the organ circulation under sterile conditions and controlled temperatures (Monbaliu and Brassil, 2010). MP devices were initially developed for kidney preservation, with research focused primarily on flow and pulsatile pressures under hypothermic (4°C) conditions. These devices used standard, non-oxygenated preservation solutions, such as UW, for perfusate (Daemen et al., 1997). The first device approved in the US by the FDA for kidney preservation in 2009 perfused the organs at 4°C without oxygenation (Moers et al., 2009). Unfortunately, this generation of devices had limited impact in both graft and patient survival 5 years after transplantation and became obsolete (De Deken et al., 2016). Subsequent MP devices were developed that instead preserved organs under normothermic (37°C) conditions and used oxygen carrier solutions for perfusate. Recently, two new MP devices have been approved for lung preservation at 37°C using purged red blood cells for the primary oxygen carrier component (Cypel et al., 2009).

Machine perfusion for liver preservation was initially conceived similarly to the aforementioned kidney devices using hypothermic preservation, and the results were analogous regarding the lack of a major benefit from this technique (Guarrera et al., 2010). Additional clinical developments with MP for liver preservation have utilized a short period of hypothermic (4°C) oxygenation (HOPE), with results exceeding initial expectations (Dutkowski et al., 2014). Most recently, a clinical trial of an MP system using fully oxygenated, normothermic red blood cells has been completed with somewhat disappointing results (Bral et al., 2016). We recently added to this growing body of work with the first application of a MP protocol in which liver allografts were fully oxygenated, under dual pressures and subnormothermic conditions (21°C), with a new HBOC solution specifically developed for *ex vivo* utilization. A comprehensive study of transcriptomic, metabolomic, histologic, and inflammatory responses highlighted multiple benefits of our MP protocol when compared to CSP (Fontes et al., 2015).

The inflammatory mediator analyses in our study suggested that perfusion with MP results in decreased tissue levels of IFN-α, IFN-γ, TNF-α, IL-1β, IL-4 and IL-12/IL23 (p40) compared to CSP, suggesting a broad-based down-regulation of the pro-inflammatory response (Fontes et al., 2015). However, inflammation is much more than single mediators. Inflammation comprises complex dynamic networks that feature hundreds of mediators from differing cell types, variability over time, and interrelation of mediators due to feedback mechanisms. Compounding this complexity is frequent pleiotropy and redundancy, as well as the multiscale aspect inherent in a system that affects multiple tissues and organs. We and others have been able to gain insights into these networks using quasi-mechanistic data-driven computational modeling based on tools such as principal component analysis and various forms of DyNA (Mi et al., 2011; Azhar et al., 2013; Vodovotz and Billiar, 2013; Ziraldo et al., 2013; Emr et al., 2014; Zaaqoq et al., 2014; Almahmoud et al., 2015; Sadowsky et al., 2015). In the work presented herein, we use Dynamic Bayesian Network (DyBN) inference and DyNA to further clarify and compare the patterns of inflammation resulting from both CSP and MP, in an effort to better understand the mechanisms by which our protocol exerts its effects.

## Materials and Methods

The experiments generating the data for this mathematical analysis were previously published (Fontes et al., 2015). Thus, the comprehensive and specific description of procedural methods can be found in the original publication. An overview of these methods has been restated below as reference for the computational analysis performed in the present study. The protocol was approved by the University of Pittsburgh Institutional Animal Care and Use Committee (IACUC, protocol number 13102322) and conducted according to the NIH Guide for the Care and Use of Laboratory Animals.

### Surgical Procedures

Allografts from two groups of six animals (Landrace pigs, 60 kg) were recovered under general anesthesia, procured according to standard surgical techniques, and flushed simultaneously through the HA and PV with 5 L UW at 4°C immediately after cross clamp and exsanguination.

The CSP allografts were preserved under standard hypothermic and static conditions with continuous temperature monitoring.

An MP system (Liver Assist^CE^) from Organ Assist (Groningen, the Netherlands) was utilized. MP livers had both arterial and PV cannulas inserted before placement into the device, with cannulation taking 15 min. The perfusate was oxygenated continuously (FiO_2_ = 60% @ 800 mL/min, O_2_ delivery = 14.1 mL O_2_/min) through microporous oxygenators. MP was conducted under low flows (PV flow = 259 ± 40 mL/min; HA flow = 91 ± 36 mL/min), stable temperature (21°C) and low pressures (PV = 3.5 ± 0.5 mmHg, HA = 18 ± 2 mmHg). Pressures were established and maintained in a stable and narrow range (Supplementary Figure S1). These values were selected after extensive prior titration studies, and are lower than physiologic values in order to avoid endothelial cell damage within organ vasculature.

A new, cell-free HBOC solution was developed by combining a stable, second-generation, bovine-derived hemoglobin compound (Jahr et al., 2008) (Hemopure^TM^; OPK Biotech, Cambridge, MA, USA) with a hetastarch-based colloid (Belzer Machine Perfusion Solution, Preservation Solutions, Inc., Elkhorn, WI, USA). The HBOC component had an initial hemoglobin concentration of 13 g/dL, a half-life of 20 h and a clearance of 0.12 L/h. This solution can bind up to 1.36 mL O_2_ per gram of hemoglobin when fully saturated (Jahr et al., 2007; McNeil et al., 2011). The combined solution (Vir1; VirTech Bio Inc., Beverly, MA, USA) had a final hemoglobin concentration of 3.5g/dL and the following characteristics: pH 7.62, osmolality = 296 mOsm/kg, colloid osmostic pressure = 59.1 mmHg, Na^+^ = 105 mmol/L, K^+^ = 17.3 mmol/L, Cl^-^ = 36 mmol/L and Ca^++^ = 0.24 mmol/L.

### Measurements

Samples of perfusate and tissue were taken at four time points: a baseline with normal blood flow was taken immediately before cross clamp application in the donor, and after 3, 6, and 9 h of preservation, either by CSP or MP as detailed above.

Tissue and perfusate assays of IFN-α, IFN-γ, IL-10, IL-12/IL-23 p40, IL-1β, IL-4, IL-6, IL-8 and TNF-α were carried out using a Luminex^TM^ beadset from Affymetrix (Santa Clara, CA, USA). GM-CSF, IL- 1α, IL-1RA, IL-2 and IL-18 were measured using a Luminex^TM^ beadset from Millipore (Merck KGaA, Darmsdadt, Germany) (Barclay et al., 2008). Perfusate samples were also assessed for AST and ALT. Tissue samples were normalized by protein content to account for experimental variability in cell number and protein concentration among individual samples. Perfusate assays were unable to be obtained for one pig in the CSP group, which was excluded from the subsequent perfusate analyses for that group.

### Dynamic Bayesian Network Inference

Given time-series data, DyBN inference is a method for suggesting causal relationships among variables based on probabilistic measure. In a DyBN, variables are shown as nodes and the interconnections are shown as edges. Unlike standard correlative approaches, DyBNs consider the joint distribution of the entire data set when making inferences about the dependencies among variables or nodes in the network. This analysis was carried out in MATLAB (The MathWorks, Inc, Natick, MA, USA), using an algorithm adapted from Grzegorczyk and Husmeier (Grzegorczyk and Husmeier, 2011) and revised by our group (Mi et al., 2011; Azhar et al., 2013; Ziraldo et al., 2013; Emr et al., 2014; Namas et al., 2014; Zaaqoq et al., 2014; Almahmoud et al., 2015; Sadowsky et al., 2015). The inference procedure was run individually for each pig, and the marginal edge probabilities averaged across all runs. The thickness of edges was weighted by this number, and only edges with an averaged marginal edge probability greater than 0.5 were included in the final consensus network for each condition. Exact edge probabilities for resultant networks are included in **Supplementary Table S1**. We note that this algorithm is unable to determine directly the positive or negative nature of edges connecting the nodes, as well as whether edges represent a linear or other type of relationship.

### Dynamic Network Analysis

After using DyBN to outline the patterns and relationships prevalent throughout the entire experimental course, DyNA was performed to track the progression of inflammatory networks during the process of organ preservation. The mathematical formation of this method is essentially to calculate the correlation among the variables by which we can examine their dependence, and we have applied it in the past to the study of inflammatory networks in murine and human data (Mi et al., 2011; Ziraldo et al., 2013; Namas et al., 2014). Networks were created in adjacent 3-h intervals (Baseline – 3 h, 3 – 6 h, 6 – 9 h). In order to be included in a network, a given mediator had to be statistically significantly different from its baseline value (*p* < 0.05 by Student’s *t*-test). Unlike DyBN, DyNA allows for the discrimination of positive and negative connections between variables. Positive edges in the network were created if the value of the correlation coefficient between two nodes (inflammatory mediators) was greater than or equal to a threshold of 0.7 (*p* < 0.05), and negative edges were created if the coefficient was less than or equal to -0.7 (*p* < 0.05). All work was carried out using MATLAB^®^ and Inkscape^1^ software. For the network density calculation we utilized the following formula (a minor revision of that reported by Assenov et al. (2008)):

$$\frac{\mathrm{Total}\;\mathrm{number}\;\mathrm{of}\;\mathrm{edges}\;*\;\mathrm{Number}\;\mathrm{of}\;\mathrm{total}\;\mathrm{nodes}}{\mathrm{Maximum}\;\mathrm{possible}\;\mathrm{edges}\;\mathrm{among}\;\mathrm{total}\;\mathrm{nodes}}$$

## Results

For each preservation method, we first sought to identify the dynamic inflammatory network characterizing the entire experimental time course, as well as identifying potential central nodes (i.e., nodes that exhibit self-feedback and that therefore may be central regulators of the dynamic inflammatory response), by using DyBN on data from all time points. DyBN depicts the entire time progression of a given network as a single graph. Then, using DyNA, we sought to define the time-dependent progression of these inflammation networks in a more granular fashion over the period of preservation, allowing for a higher resolution portrayal of the time course of inflammation as well as highlighting both positive and negative regulatory interactions. The tradeoff in this analysis is that DyNA, unlike DyBN inference, does not define self-feedback structures. Analyses were completed on both tissue and perfusate data to allow for multiple representations of the liver.

**FIGURE 1:**
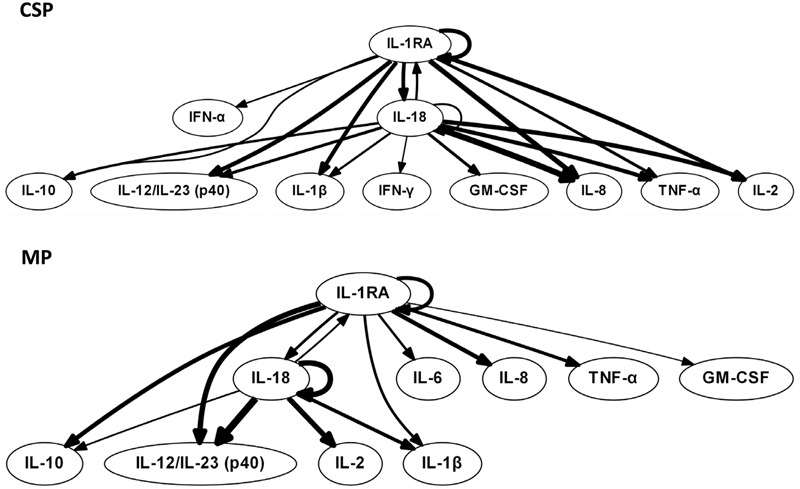
**Tissue sample DyBNs suggest conserved network structure with central nodes IL-18 and IL-1RA, but decreased pro-inflammatory response with MP.** CSP, cold static preservation (*n* = 6). Organs were preserved at standard hypothermic and anoxic conditions. MP, machine perfusion (*n* = 6). Organs were fully oxygenated under dual pressures under subnormothermic conditions with a new hemoglobin-based oxygen carrier solution specifically developed for *ex vivo* utilization. Multiple inflammatory mediators were interrelated using DyBN inference. While the overall network structure is maintained, MP features fewer edges from the pro-inflammatory IL-18 than CSP.

**FIGURE 2:**
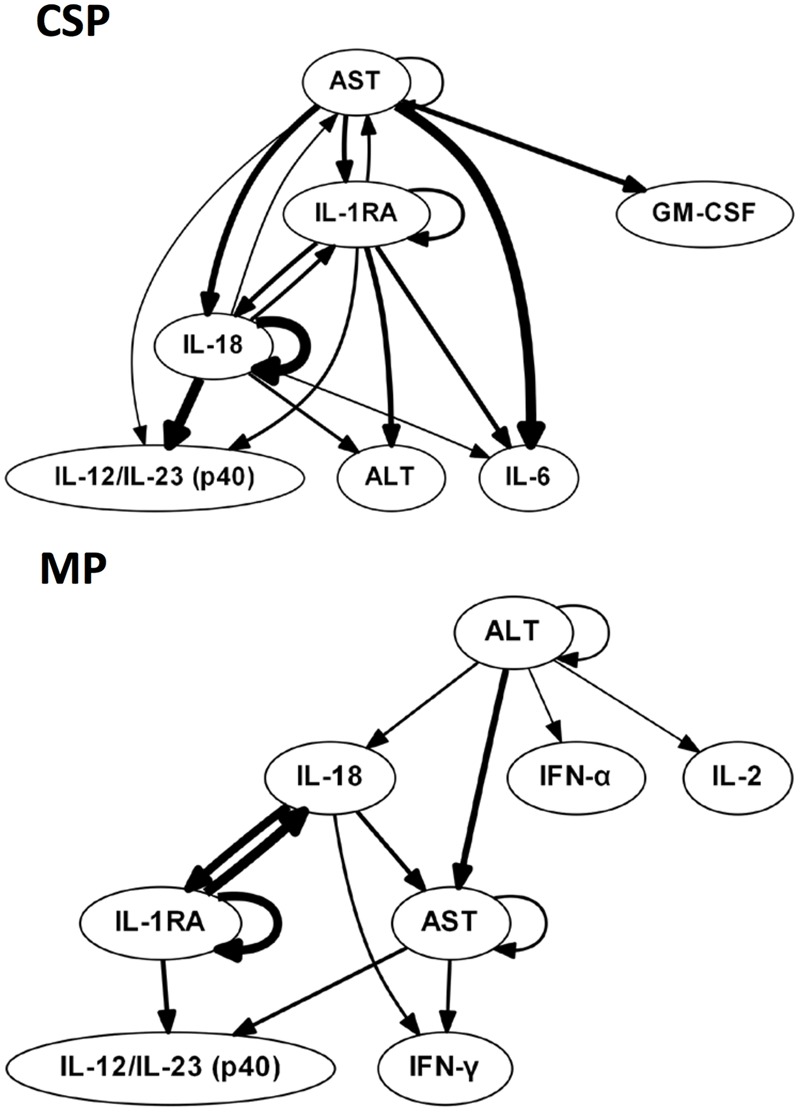
**Perfusate sample DyBNs suggest conserved network structure with central nodes IL-18 and IL-1RA, but decreased pro-inflammatory response and liver damage with MP.** CSP, cold static preservation (*n* = 5). Organs were preserved at standard hypothermic and anoxic conditions. MP, machine perfusion (*n* = 6). Organs were fully oxygenated under dual pressures under subnormothermic conditions with a new hemoglobin-based oxygen carrier solution specifically developed for *ex vivo* utilization. Multiple inflammatory mediators and liver damage markers AST and ALT were interrelated using DyBN inference. While the overall network structure is maintained, MP features fewer edges from the pro-inflammatory IL-18 than CSP, including a loss of both IL-18 self-feedback and an edge from IL-18 to major pro-inflammatory mediator IL-6. The centrality of liver damage markers AST and ALT is also decreased with MP, as seen most notably by the loss of bidirectional edges between AST and both IL-18 and IL-1RA.

We hypothesized that central nodes (which we define as DyBN network nodes that exhibit self-feedback and that connect to other nodes that do not have this property) represent key inflammation control mechanisms. Our analyses of tissue data (**Figure 1**) suggest an overall response coordinated by the pro-inflammatory cytokine IL-18 and the anti-inflammatory IL-1 receptor antagonist (IL-1RA). Of note, treatment with MP is associated with an appreciably diminished centrality of IL-18 as compared to CSP, with IL-18 having only 4 external connections (not to itself or to IL-1RA) in the MP network compared with 8 outputs in the CSP network. Our analyses of perfusate data (**Figure 2**) also infer networks driven by IL-18 and IL-1RA, with additional central nodes including AST in the CSP group vs. AST and ALT in the MP group. Again, in these analyses we infer a reduced centrality of IL-18 in the MP group as compared to the CSP group, with one fewer output and no self-feedback in the MP network.

The liver injury marker AST is also less central in the MP perfusate network, similar to what we infer for IL-18, with only 2 non-self outputs compared to 5 in CSP. Additionally, the CSP network includes bidirectional edges between AST and both IL-18 and IL-1RA, while the MP network only contains a unidirectional edge to AST from IL-18 and no edges between AST and IL-1RA. Another noteworthy difference is the output edge of AST to IL-6 in the CSP network only, with IL-6 not present at all in the MP network.

Tissue DyBNs show more connections from IL-1RA to pro-inflammatory mediators in MP networks than CSP. For example, an output from IL-1RA to the major pro-inflammatory mediator IL-6 only exists in the MP network. As noted in “*Materials and Methods*,” edges between nodes in DyBNs may be of a positive or negative nature. Given the known role of IL-1β as an inducer of IL-6, we hypothesize that edges from IL-1RA to other inflammatory mediators represent a negative effect, something that is not observed in CSP. Similarly, other pro-inflammatory mediators that appear in both tissue networks (IL-8, TNF-α, and GM-CSF) are only connected to IL-1RA in MP, but connect to both IL-1RA and IL-18 in CSP.

**FIGURE 3:**
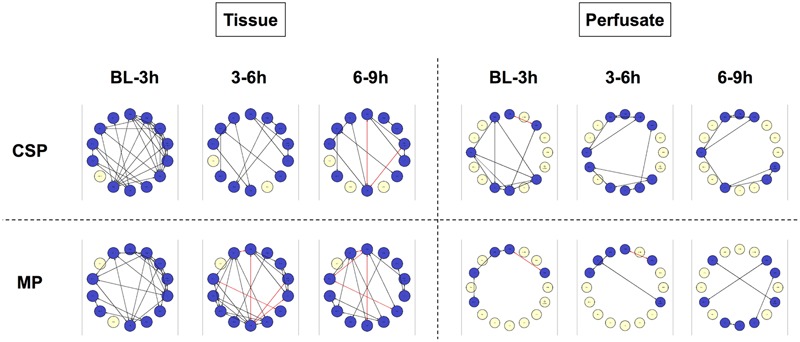
**Tissue sample DyNA networks contain more variables, total and negative edges than perfusate.** CSP, cold static preservation (*n* = 6, tissue; *n* = 5, perfusate). Organs were preserved at standard hypothermic and anoxic conditions. MP, machine perfusion (*n* = 6, tissue; *n* = 6, perfusate). Organs were fully oxygenated under dual pressures under subnormothermic conditions with a new hemoglobin-based oxygen carrier solution specifically developed for *ex vivo* utilization. DyNA networks were then constructed for all timeframes, with variables and edges included in each network. Tissue networks consistently include more variables and connections than perfusate. Tissue networks also feature numerous negative (red, anticorrelated) edges. Within the tissue analyses, MP networks contain more negative edges and feature the earlier appearance of negative edges than CSP.

**FIGURE 4:**
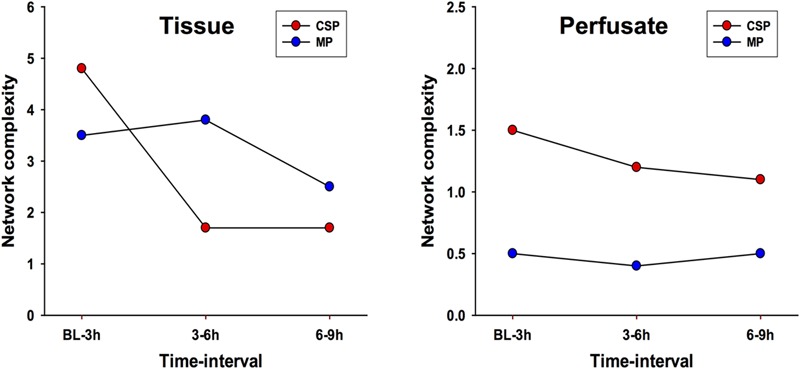
**Network complexity DyNA.** CSP, cold static preservation (*n* = 6, tissue; *n* = 5, perfusate). Organs were preserved at standard hypothermic and anoxic conditions. MP, machine perfusion (*n* = 6, tissue; *n* = 6, perfusate). Organs were fully oxygenated under dual pressures under subnormothermic conditions with a new hemoglobin-based oxygen carrier solution specifically developed for *ex vivo* utilization. Network complexity scores are consistently higher in tissue networks than perfusate. For both sample types, the initial CSP network is the most complex, with CSP network complexity decreasing over time. MP networks, which for both sample types start with lower complexity than CSP, remain relatively static in complexity throughout the preservation period.

DyNA networks were then constructed for all time intervals, with variables and edges included in each network (**Figure 3**). Tissue networks consistently include more variables and connections than perfusate. Tissue networks also feature numerous negative (anticorrelated) edges, which we interpret as suppressive interactions. Within the tissue analyses, MP networks contain more negative edges and feature the earlier appearance of negative edges than CSP. Network complexity scores (**Figure 4**) are consistently higher in tissue networks than perfusate. For both sample types, the initial CSP network is the most complex, with CSP network complexity decreasing over time. MP networks, which for both sample types start with lower complexity than CSP, remain relatively static in complexity throughout the preservation period.

**FIGURE 5:**
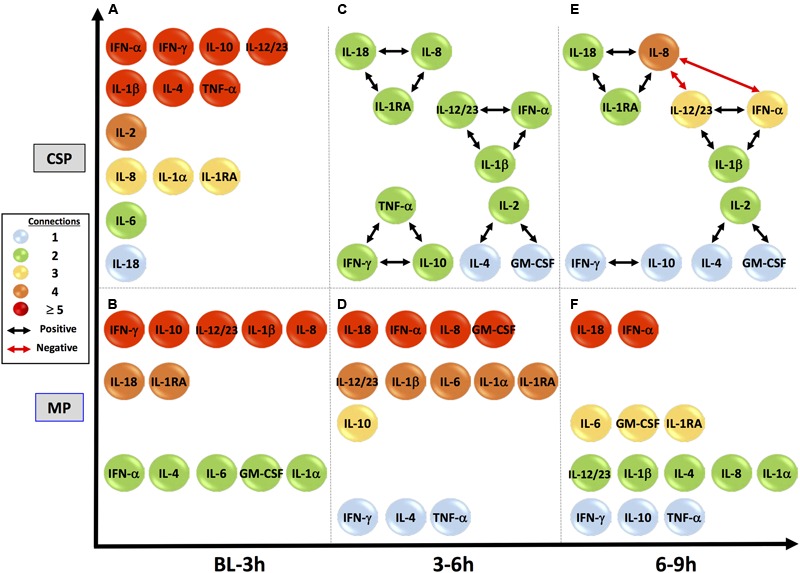
**Tissue sample DyNA networks suggest different network stability between preservation methods.** CSP, cold static preservation (*n* = 6). Organs were preserved at standard hypothermic and anoxic conditions. MP, machine perfusion (*n* = 6). Organs were fully oxygenated under dual pressures under subnormothermic conditions with a new hemoglobin-based oxygen carrier solution specifically developed for *ex vivo* utilization. IL-18, IL-1β, IL-1RA and IFN-γ are among the variables included in every tissue DyNA network. In the CSP analysis a complex initial network is followed by two networks in which a majority of variables only have two edges and whose structures are almost identical. Unlike CSP, the MP networks continue to fluctuate throughout the experimental time course.

**FIGURE 6:**
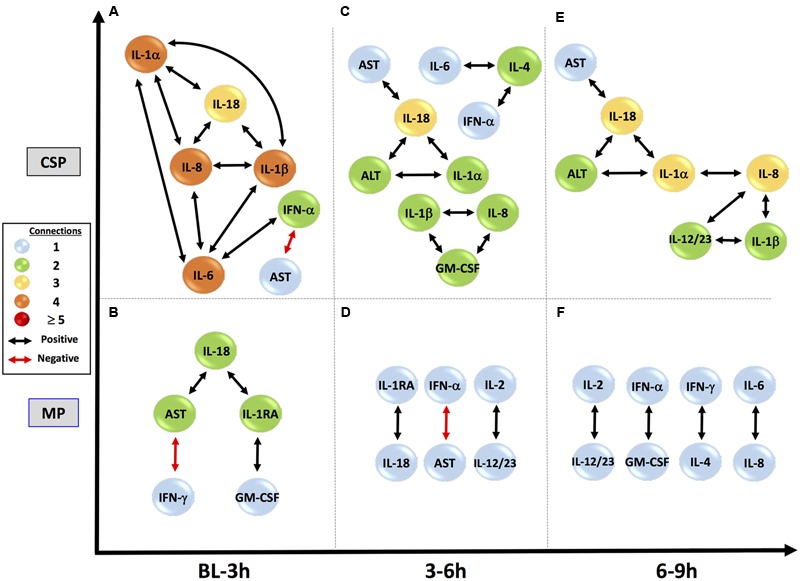
**Perfusate sample DyNA networks suggest divergent responses to preservation methods.** CSP, cold static preservation (*n* = 5). Organs were preserved at standard hypothermic and anoxic conditions. MP, machine perfusion (*n* = 6). Organs were fully oxygenated under dual pressures under subnormothermic conditions with a new hemoglobin-based oxygen carrier solution specifically developed for *ex vivo* utilization. CSP networks show initial inclusion and interrelation of numerous pro-inflammatory mediators, including IL-18, and progress to stable connections between IL-18 and both AST and ALT, suggesting continual inflammation-driven liver damage. Anti-inflammatory IL-1RA never appears. MP networks show a restrained initial network, with IL-18 connected to IL-1RA and damage marker AST, but not to pro-inflammatory mediators. IL-18 then loses its connection to AST and by the final time period IL-18, IL-1RA, AST and ALT are not included, suggesting control and resolution of inflammation with minimal liver damage.

IL-18, IL-1β, IL-1RA and IFN-γ are among the variables included in every tissue DyNA network (**Figure 5**). In the CSP analysis, a complex network for the initial timeframe is followed by two networks in which a majority of variables only have two edges. Of note, these latter networks are almost identical, with the only differences being two additional edges connecting IL-8 with IL-12/23 (p40) and IFN-α, along with the absence of TNF-α in the final network. Unlike CSP, the MP networks continue to fluctuate throughout the experimental time course, suggesting the possibility that MP actively and repeatedly counters inflammatory signaling. A similar pattern is seen the perfusate DyNA networks (**Figure 6**). The CSP analysis again features an initially complex network followed by two simpler and similar networks. Likewise, the MP analysis again features fluctuating networks throughout the preservation period.

In the early time interval of DyNA networks inferred in perfusate obtained from CSP organs (**Figure 6A**), IL-18 is connected to multiple pro-inflammatory mediators (IL-1α, IL-1β and IL-8, which all then connect to IL-6). IL-1RA is not included in this network. In MP organs during this interval (**Figure 6B**), however, IL-1RA is included and connects to IL-18, which has no connections to other inflammatory mediators.

Subsequent perfusate DyNA networks suggest that the responses in CSP and MP organs continue to diverge as time spent under preservation progresses. By 3–6 h in the CSP group, IL-18 has developed direct connections to the liver damage markers AST and ALT, and these connections continue in the 6–9 h interval (**Figures 6C,E**). IL-1RA never appears in the CSP networks. The 3–6 h interval in MP (**Figure 6D**) continues to show the connection between IL-1RA and IL-18 seen initially, but has lost the connection between IL-18 and AST from the first interval. By 6–9 h (**Figure 6F**), IL-18, IL-1RA, and AST/ALT are no longer present in the network (meaning they are no longer significantly different from their baseline values).

Taken together, we interpret the CSP and MP results to suggest that IL-18 (or pathways responsible for the generation of IL-18, such as the NLRP3 inflammasome; Elliott and Sutterwala, 2015) drives multiple secondary inflammatory mechanisms triggered by IR insult that subsequently or consequently lead to liver injury. In CSP, this process is persistent and sometimes (67% mortality) irreversible, whereas in MP this process is attenuated and eventually blocked by what we interpret to be an IL-1RA driven anti-inflammatory response. In perfusate, we observed significantly higher levels of IL-1RA with MP vs. CSP (**Table 1**). Notably, these inferences of a central role for IL-18 and a diminished role for IL-18 in MP vs. CSP livers occur despite a lack of statistically significant differences in the levels of IL-18 between protocols for either sample type; likewise, there is no statistically significant difference in the tissue levels of IL-1RA analyzed in our original work (**Table 1**).

**Table 1:** Perfusate IL-1RA levels are significantly lower with CSP vs. MP.

Sample Type	Cytokine	Protocol	Mean ± SEM, pg/mL	*P* Value
Perfusate	IL-18	CSP	738 ± 111	0.299
		MP	932 ± 155	
	IL-1RA	CSP	230 ± 34	0.005^∗^
		MP	7317 ± 1953	
Tissue	IL-18	CSP	1600 ± 153	0.839
		MP	1544 ± 243	
	IL-1RA	CSP	2478 ± 270	0.539
		MP	2733 ± 324	

*CSP, cold static preservation (Perfusate, *n* = 5; Tissue, *n* = 6). Organs were preserved at standard hypothermic and anoxic conditions. MP, machine perfusion (Perfusate, *n* = 6; Tissue, *n* = 6). Organs were fully oxygenated under dual pressures under subnormothermic conditions with a new hemoglobin-based oxygen carrier solution specifically developed for *ex vivo* utilization. Two-way ANOVA was performed on cytokine levels for 3, 6, and 9 h of preservation. There is no statistically significant difference in the tissue levels of either cytokine analyzed in our original work. ^∗^statistically significant.*

## Discussion

The preservation of organs undergoing transplantation has recently become a focus of research seeking to improve organ availability and clinical outcomes (Ali et al., 2015). Our prior work demonstrated the suppression of numerous pro-inflammatory mediators by MP compared to CSP, implying a decreased inflammatory response associated with improved liver preservation. These measurements were accompanied by transcriptomic, metabolomic, histologic and clinical results largely supporting a conclusion of improved organ and systemic function associated with MP (Fontes et al., 2015). In the present study, we hypothesized that different, dynamic networks of inflammation characterize – and likely play a key role in – the differential impact of MP vs. CSP on the preservation process.

Whereas traditional statistical approaches are able to identify and determine significant differences in levels of various variables, DyBN inference and DyNA allow us to better understand subtle relationships among variables (beyond the broad types of changes that are defined by traditional statistical analyses). Defining dynamic networks also allows us to form novel hypotheses regarding key control mechanisms. We have previously demonstrated the use of DyBN inference in assessing systemic inflammatory networks in human liver transplant patients (Azhar et al., 2013) and on porcine data in studying responses to acute trauma and sepsis, including a model incorporating multiple sample types representative of discrete bodily compartments (Emr et al., 2014; Sadowsky et al., 2015). Our work with DyNA demonstrated novel features associated with both experimental and clinical trauma/hemorrhage (Mi et al., 2011; Ziraldo et al., 2013; Namas et al., 2014), as well as defining networks of inflammatory mediators in murine hepatocytes exposed to hypoxia (Ziraldo et al., 2013). We have now applied these methods to the study of liver preservation, in the context of tissue and perfusate samples from livers subjected to CSP vs. MP.

We found similar results in both liver tissue and perfusate samples. Our studies suggest that the resultant network structures from DyBN inference, which encompass all data from the complete duration of the experiment, are preserved across sample types. This similarity is to be expected given the related nature of liver tissue and perfusate, shows that perfusate (representing the intravascular and extracellular space) can be used as reflection of processes occurring in tissue, and serves as an internal demonstration of the consistency and reproducibility of our methods and results. Our results suggest that perfusate could be analyzed in serial fashion, pointing to practical applications of our study: perfusate is also more readily obtainable than tissue, and unlike tissue the process of sampling it does not induce injury. Our results thus suggest that defining dynamic networks of inflammation and organ (patho)physiology based on data obtained from the perfusate can serve as a potential diagnostic strategy.

Our analytical strategy may also help elucidate novel mechanistic interactions. In the present study, the inferences obtained by our algorithms correlate well with both known and novel inflammatory and hepatic pathophysiology centered on the inflammasome pathway (Hoque et al., 2013). IL-18 is a product of the NLRP3 inflammasome, a multi-enzyme complex that also leads to the production of mature IL-1β (Elliott and Sutterwala, 2015), and is a potent pro-inflammatory cytokine that exerts its effects early in inflammatory pathways (Tsutsui et al., 2000; Gracie et al., 2003). While not specific to the liver, IL-18 expression is known to be enhanced in acute hepatic diseases such as fulminant hepatic failure and acute hepatitis (Yumoto et al., 2002). The NLRP3 inflammasome is expressed in the liver by KC, including in hepatic I/R injury (Kim et al., 2015). KC are the key cell type in early hepatic injury, and the key mediator in this process is IFN-γ (Weigand et al., 2012). In response to hepatic insult, IL-1β and IL-18 are released by KC via NLRP3. IL-1β, in addition to its downstream pro-inflammatory effects, recruits CD4^+^ T cells to the liver, and these CD4^+^ T cells in turn release IFN-γ (Weigand et al., 2012). IL-18, meanwhile, directly up-regulates Natural Killer-T cells (NKT), which are major producers of IFN-γ (Okamura et al., 1998; Tsutsui et al., 2000). IL-18 also acts synergistically with IL-12 to stimulate IFN-γ production in CD4^+^ T cells, having little effect alone on this particular cell type (Tsutsui et al., 2000). IFN-γ further damages hepatocytes and stimulates KC, creating a positive feedback loop that drives this process and makes IFN-γ a key determinant in the overall strength of response (Abu-Amara et al., 2010; Weigand et al., 2012). The NLRP3 inflammasome is expressed elsewhere in the liver as well (including sinusoidal endothelial cells, portal fibroblasts, stellate cells and hepatocytes), though levels are highest in KC (Boaru et al., 2012).

The primary negative regulator of the NLRP3-driven inflammatory response is IL-1RA, which prevents the binding of IL-1β to its receptor and therefore blocks it from exerting its effects. Hepatocytes are the primary source of IL-1RA during sterile inflammation (Lamacchia et al., 2010), and this protein is an acute phase reactant (Gabay et al., 1997) that responds to IL-1β (Lamacchia et al., 2010), including in humans (Bargetzi et al., 1993). Indeed, treatment with IL-1RA reduces hepatic inflammation in an *in vitro* model of hyperactive NLRP3 (Wree et al., 2014).

Much of this well-described response is captured in our results. The early and central role for the NLRP3 inflammasome is represented by the centrality and upstream nature of IL-18 and IL-1RA, the latter of which, as noted in the *Results*, we believe is directly reflective in our networks of the function of IL-1β. The output of IFN-γ in response to NLRP3 activation is seen as well: two of four DyBNs contain an edge from IL-18 to IFN-γ, thus inferring correctly the major downstream effect of IL-18 in this system. Additionally, all DyBNs except one capture the connection between IL-18 and IL-12/IL-23 (p40). Interestingly, the only DyBN missing IL-18 self-feedback is also the only one that lacks an edge between IL-18 and IL-12/IL-23 (p40), further reinforcing the possible synergy between these two cytokines in the production of IFN-γ. Finally, a role for liver damage (as represented by AST and ALT) in driving further inflammation is also implied by DyBN inference: AST self-feedback in perfusate networks. The direct connection of IL-18 to this damage is suggested by the inclusion of both an input and output edge between IL-18 and at least one damage marker in perfusate DyBNs, and by edges between IL-18 and AST/ALT in multiple CSP perfusate DyNA networks.

**FIGURE 7:**
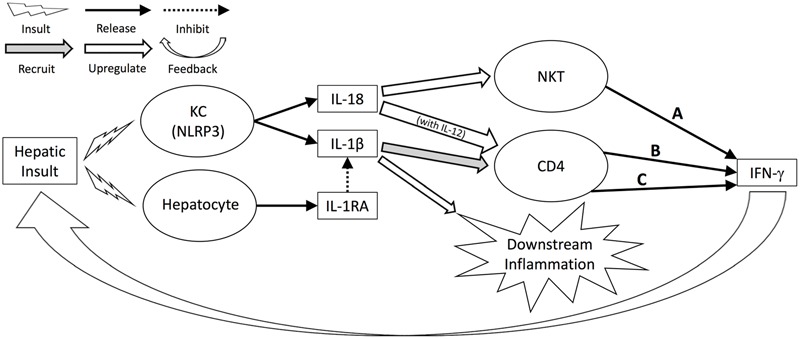
**Hepatic response to insult regulated by IL-18, IL-1RA.** Levels of IFN-γ, and by extension the strength of the IFN-γ-driven positive feedback loop of hepatic damage, are connected in three pathways to the early, NLRP3-driven hepatic response to insult. One of these pathways can be downregulated by IL-1RA (C), one can be upregulated by IL-18 (A), and one can be both downregulated by IL-1RA and upregulated by IL-18 (B). Thus, the relative balance of these two mediators may control the level of response.

Our results, in addition to capturing known responses, may also help to explain the mechanisms by which MP leads to improved outcomes. As described above and summarized in **Figure 7**, levels of IFN-γ, and by extension the strength of the IFN-γ-driven positive feedback loop of hepatic damage, are connected in three pathways to the early, NLRP3-driven hepatic response to insult. One of these pathways can be downregulated by IL-1RA (C), one can be upregulated by IL-18 (A), and one can be both downregulated by IL-1RA and upregulated by IL-18 (B). Thus, the relative balance of these two mediators may control the level of response. DyBN inference suggests a decreased IL-18-driven response, increased IL-1RA-driven response, and decreased liver damage with MP compared to CSP.

DyNA results for both tissue and perfusate analyses show MP networks that fluctuate in complexity and pattern throughout the preservation period, while CSP networks stay relatively constant after 3 h. We hypothesize that this difference between protocols reflects an early response in CSP organs that generates enough positive feedback to “lock” the organ into a pattern of self-sustaining inflammatory damage, while MP provides sufficient metabolic support to reduce the positive feedback loop of (IL-18-driven) inflammation → damage (AST/ALT) → inflammation, presumably through mechanisms which decrease NLRP3 activation and/or stimulate the release of IL-1RA. These results were all aligned with previously described clinical and laboratorial findings related to hepatic function during preservation and after liver allograft reperfusion (Fontes et al., 2015).

This work highlights the value that may be added by augmenting conventional statistical analyses with data-driven modeling techniques. Whereas our original results point to an overall decrease in inflammation with MP treatment based on statistical analysis of a collection of individual pro-inflammatory mediators (Fontes et al., 2015), our current work serves to elucidate the interconnected and dynamic nature of this alteration. More specifically, our current work points to differential regulation of the inflammatory response by IL-18 and IL-1RA. Importantly, the tissue levels of these mediators in our original analysis were not statistically significantly different between the CSP and MP groups. Rather, these mediators are inferred to act upstream of those significantly altered mediators, and earlier in time, creating an alternative and potentially more worthwhile direction for future experimentation. We suggest that the expanded use of computational modeling, in iteration with traditional experimentation and analysis, may be a means to accelerate and add efficiency to the study of complex inflammatory pathways.

In addition to expanding the methods available to scientists researching inflammation, our work also serves as a framework for future studies in organ preservation, which could lead into a rather important predictive tool for IR lesions sustained before, during, and after organ preservation. Because of the aforementioned comprehensive study of the same organs and data used herein, our current results can be interpreted not just as an isolated comparison of two preservation protocols, but more broadly as prototype networks representing well- vs. poorly preserved organs. With these results as a baseline, networks could be constructed using limited organs and resources, in order to screen new preservation protocols and to identify therapeutic or diagnostic candidates worthy of more extensive study. In the future, as research on discarded organs expands and preservation methods are improved, modeling may be used to characterize different disease states and track attempts to improve organ function in an extracorporeal environment.

## Conclusion

We suggest that the use of dynamic network analyses on inflammation biomarker data in the setting of liver preservation both complements our prior results and substantiates our understanding of the cellular and sub-cellular mechanisms by which MP exerts its observed beneficial effects. These computational tools may serve as a framework for discovering novel diagnostic or therapeutic modalities for enhanced liver preservation.

## Author Contributions

Conception and design of work: DS, YV, and PF. Acquisition, analysis, and interpretation of data: DS, DB, JY, and RZ. Drafting, revision, and approval of manuscript: All authors Agreement to be accountable for all aspects of presented work: All authors.

## Conflict of Interest Statement

The authors declare that the research was conducted in the absence of any commercial or financial relationships that could be construed as a potential conflict of interest.

## Footnotes

1 http://inkscape.org

## Supplementary Material

TABLE S1 **Percentage of networks in which edge is included.** These values correlate directly with edge thickness in DyBN figures.

## Abbreviations

ALT: alanine transaminase
AST: aspartate transaminase
CSP: cold static preservation
DyBN: dynamic Bayesian Network inference
DyNA: dynamic network analysis
GM-CSF: granulocyte macrophage colony-stimulating factor
HA: hepatic artery
HBOC: hemoglobin-based oxygen carrier
IFN: interferon
IL: interleukin
IR: ischemia/reperfusion
KC: Kupffer cell
MP: machine perfusion
NKT: natural killer T-cell
PaO_2_: arterial oxygen pressure
PV: portal vein
SaO_2_: arterial oxygen saturation
TNF: tumor necrosis factor
UW: University of Wisconsin solution
