# Supplementary material for: Machine Perfusion of Porcine Livers with Oxygen-Carrying Solution Results in Reprogramming of Dynamic Inflammation Networks
Source: Front Pharmacol. 2016 Nov 4;7:413. doi: 10.3389/fphar.2016.00413 (PMC5095594; doi:10.3389/fphar.2016.00413)
Supplement: Supplementary file 2 [file Data_Sheet_1.DOCX]

Supplementary Figure 1: Perfusion pressures were maintained in stable and narrow ranges. (HA 18mmHg, range 15-20; PV 3.5mmHg, range 3-4)
